# Focal Epileptiform Discharges Can Mimic Electrode Artifacts When Recorded on the Scalp Near a Skull Defect

**DOI:** 10.1177/2324709618795305

**Published:** 2018-08-21

**Authors:** Edward C. Mader, Daniella Miller, Jeremy M. Toler, Piotr W. Olejniczak

**Affiliations:** 1 Louisiana State University Health Sciences Center, New Orleans, LA, USA; 2 Children’s Hospital of New Orleans, New Orleans, LA, USA

**Keywords:** EEG, breach rhythm, electrode artifact, skull defect, 10-10 electrodes, physiological field, volume conduction

## Abstract

Breach rhythm, the hallmark of skull defect, is a familiar finding in the electroencephalogram (EEG). A hole in the skull can also give rise to unfamiliar EEG findings. We present 3 patients with a skull defect whose scalp EEG showed focal epileptiform discharges that resembled F4 electrode artifacts—a 23-year-old man with a right-sided craniectomy for traumatic brain injury, a 63-year-old woman with a history of bifrontal craniectomy and meningioma resection, and a 77-year-old woman who had a right hemicraniectomy for a life-threatening subdural hematoma. In all 3 patients, the F4 electrode was directly above or near a skull defect, and scalp EEG showed phase-reversing waves in FP2-F4 and F4-C4 with no clear-cut “physiological field” (even when the EEG was displayed at a higher sensitivity). In the first 2 patients, the technologist tried to eliminate the “electrode artifacts” by cleaning the scalp thoroughly, replacing the F4 electrode, and maintaining electrode impedance between 2 and 5 kΩ. These measures failed to eliminate the “electrode artifacts” so the EEG was recorded from four 10-10 electrode sites around F4. Extending the montage made it clear that what appeared as F4 electrode artifacts were actually focal epileptiform discharges. Correlation with other electroclinical and neuroimaging data was enough to resolve this issue in the third patient, obviating the need to extend the montage. When recording and interpreting the EEG of patients with a craniotomy or craniectomy, EEG professionals should be aware that focal epileptiform discharges can masquerade as electrode artifacts.

## Introduction

The enhanced or high-amplitude rhythmic activity seen in the electroencephalogram (EEG) of individuals with a skull defect is known as *breach rhythm*.^1,2^ A hole in the skull can modify the appearance of EEG waves by altering the propagation of EEG signals from the cortex to the scalp.^3^ During EEG signal transmission, signal loss occurs in proportion to the distance between electrodes and signal source, the electrical properties of tissues in the conductive medium, and the frequency of the signal.^4,5^ EEG signals passing through an *intact skull* are subject to the averaging and smoothing effects of bone.^5,6,7^ A *skull defect* can partially (eg, craniotomy) or completely (eg, craniectomy) abolish the smoothing effect of bone and accentuate EEG rhythms. Signal attenuation and smoothing with bone present and signal accentuation and deblurring with bone absent are both frequency-dependent, that is, the faster the EEG rhythm the more pronounced the effect.^1,8^ EEG recorded over a skull defect will show higher overall spectral power, greater amount of fast components, better interelectrode spatial resolution, and reduced artifact susceptibility compared with EEG from a homologous head region with intact skull.^3^ In essence, a skull defect provides a true “window of opportunity” to examine the EEG signal close to its source.

Electrode artifacts may appear in the EEG when the charge distribution or current flow in the electrode-scalp interface is disturbed or when current flow inside the electrode wire is altered by external fields.^9,10^ Noise may originate from the electrode-scalp interface because of electrode movement, polarization potentials, and other forms of electrochemical instability.^10,11^ Alternating current noise can enter the EEG recording system through the electrode-scalp interface because of poor electrode contact or electrode impedance mismatch or through the electrode wires via electromagnetic induction.^12^ Electrode artifacts can take the form of electrode pops, repetitive waves with variable frequencies, 60 Hz/50 Hz alternating current artifact, or continuously oscillating waves that can mimic seizures.^9^ Albeit common, electrode artifacts do not usually pose a challenge in EEG interpretation. A competent EEG technologist will immediately recognize an electrode artifact and implement measures to get rid of the artifact.^13^ Several electrode artifacts may contaminate the record, but *each electrode artifact* represents the signal input from a single electrode. Thus, in a bipolar montage, each electrode artifact typically appears as phase-reversing waves in 2 adjacent channels.^14^

Electric fields of cerebrocortical origin appear as scalp EEG potentials with a *physiological field*.^15^ A physiological field is a characteristic pattern of voltage distribution on the scalp—there is a large drop in voltage near the site of maximum voltage (resulting in a steep voltage gradient) and a much smaller drop in voltage farther away from the site of maximum voltage (resulting in a shallow voltage gradient). As a rule, EEG waves that represent cortically generated electric fields will exhibit some type of physiological field on the scalp.^14^ This is true for EEG waves that are expressed during normal brain activity (eg, alpha rhythm, vertex waves) and for EEG waves that represent neuropathological processes (eg, epileptiform spikes, polymorphic delta activity). On the other hand, electrode artifacts are not expected to exhibit a physiological field.^9,15^

EEG professionals are unlikely to misinterpret breach rhythm and electrode artifacts, both of which are familiar EEG findings. Nevertheless, the following 3 cases will illustrate that a skull defect may modify the scalp EEG appearance of cortically generated epileptic discharges to the extent that focal epileptiform discharges no longer exhibit a physiological field and can be mistaken for electrode artifacts on a standard bipolar montage.

## Case Presentation

These 3 cases make up all the cases we have encountered in which a skull defect resulted in focal epileptiform discharges that mimicked electrode artifacts in the EEG. Since we became aware of this phenomenon only 3 years ago (during our experience with patient 1), it is possible that we have overlooked other cases in the past. We will limit our presentation to information that is relevant to the topic. Note that the involvement of the F4 electrode in all 3 patients is purely coincidental.

**Figure 1.**
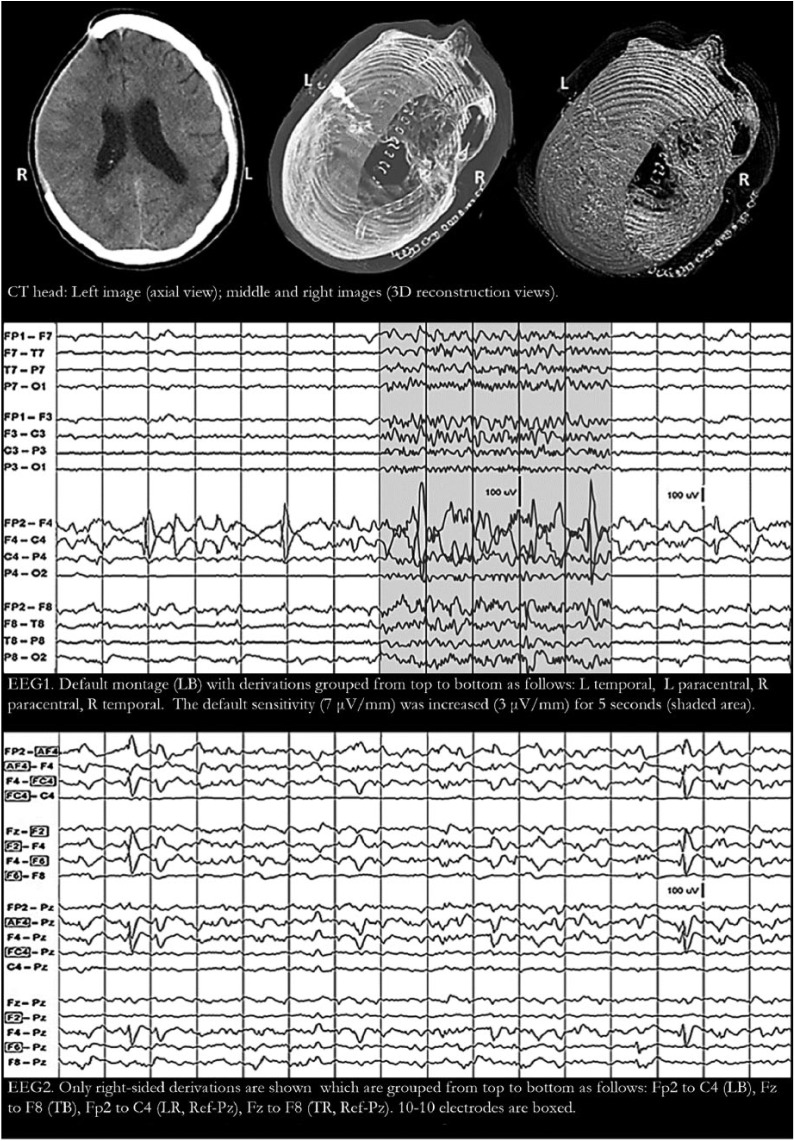
Patient 1 has a large right frontotemporal craniectomy with absent bone flap (CT [computed tomography] head). Electroencephalogram (EEG) monitoring showed phase reversals in FP2-F4 and F4-C4 consistent with F4 electrode artifacts (EEG1). Increasing display sensitivity showed an occasional hint of a physiological field but this was not enough to rule out electrode artifact (EEG1 shaded area). Transferring 4 electrodes from the left side of the head to 10-10 electrode locations around F4 (AF4, FC4, F2, and F6) made it clear that the F4 potentials have some type of physiological field (EEG2). For all EEG tracings above: filters at 1-Hz high-pass, 70-Hz low-pass, and 60-Hz notch; 1 second/division; and100-µV voltage scale shown in tracing. L, left; R, right; Ref, reference; LB, longitudinal bipolar; TB, transverse bipolar; LR, longitudinal referential; TR, transverse referential.

Patient 1 is a 23-year-old man who presented in convulsive status epilepticus. He suffered traumatic brain injury and had a right frontal craniectomy 5 months prior to admission; bone flap replacement was delayed due to hospital-acquired infection. Computed tomography (CT) head showed the expected skull defect and old lesions (Figure 1: CT head). Since lorazepam, levetiracetam, and lacosamide did not abort the seizures, he was intubated and propofol was started. EEG monitoring showed high-amplitude waves with phase reversals in FP2-F4 and F4-C4 F4. Because these waves did not show a clear-cut “physiological field”—even when display sensitivity was increased from 7 to 3 µV/mm—they were thought to be F4 electrode artifacts (Figure 1: EEG1). Carefully cleaning the scalp, replacing the electrodes, and keeping electrode impedances between 2 and 5 kΩ failed to eliminate the “electrode artifacts.” Switching to a transverse bipolar and a referential montage did not help clarify the issue. Thus, 4 electrodes were removed from the left side of the head (spare electrodes were not within reach) and attached to 10-10 locations around F4 (AF4, FC4, F2, F6). An extended montage was then constructed (Figure 1: EEG2). This simple maneuver proved that what appeared as F4 electrode artifacts were actually focal epileptiform discharges with an exceptionally “compact” electric field, that is, voltage drop was rapid at short distances from the peak.

**Figure 2.**
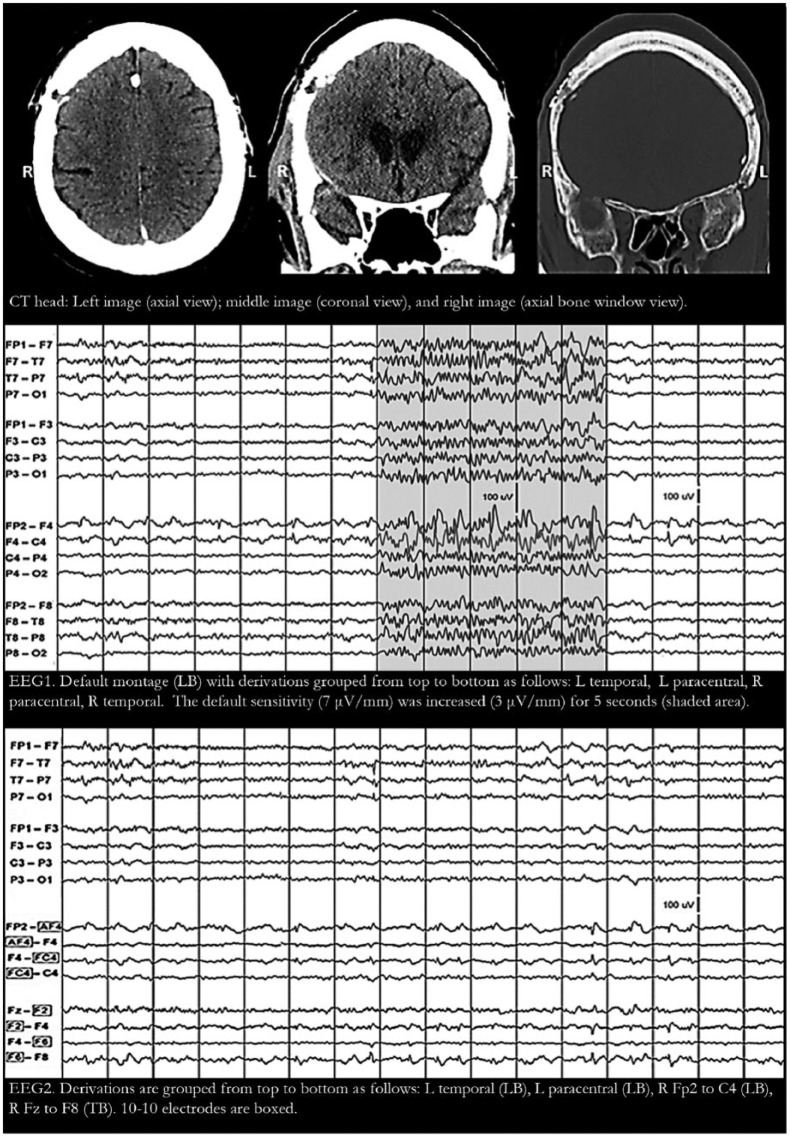
Patient 2 has residual skull holes in the frontal area from a meningioma resection surgery (CT [computed tomography] head). Electroencephalogram (EEG) showed epileptiform spikes in T3 and F7 (both with a clear-cut physiological field) and phase reversals in FP2-F4 and F4-C4 consistent with an F4 electrode artifact (EEG1). Increasing display sensitivity showed an occasional hint of a physiological field but this was not enough to rule out electrode artifact (EEG1 shaded area). Adding 10-10 electrodes (AF4, FC4, F2, and F6) and extending the montage showed that the F4 potentials have a physiological field (EEG2). For all EEG tracings above: filters at 1-Hz high-pass, 70-Hz low-pass, and 60-Hz notch; 1 second/division; and 100-µV voltage scale shown in tracing. L, left; R, right; Ref, reference; LB, longitudinal bipolar; TB, transverse bipolar; LR, longitudinal referential; TR, transverse referential.

Patient 2 is a 63-year-old woman who arrived in the emergency room in a state of delirium. She was on divalproex and zonisamide for seizure disorder, which started after resection of a frontal meningioma. CT head showed a skull defect overlying a right frontal lobe encephalomalacia (Figure 2: CT head). In addition to clear-cut epileptiform spikes in T3 and F7, EEG showed phase-reversing sharp and slow waves in Fp2-F4 and F4-C4 with no clear-cut “physiological field”—even when display sensitivity was increased from 7 to 3 µV/mm (Figure 2: EEG1). As in patient 1, troubleshooting the electrodes and montage reformatting were performed, but we were able to conclude that the F4 potentials are focal epileptiform discharges only when additional 10-10 electrodes were placed on the head (Figure 2: EEG2). Spare electrodes were immediately available (unlike the first case) obviating the need to remove electrodes already attached to the head. Extending the montage proved that the F4 phase reversals were focal periodic epileptiform discharges. Because of the exceptionally focal scalp electric field, F4 was the only 10-20 electrode detecting a scalp potential. Adding 10-10 electrodes and extending the montage allowed us to “see” a physiological field that was “invisible” when the EEG was recorded from 10-20 electrodes only.

**Figure 3.**
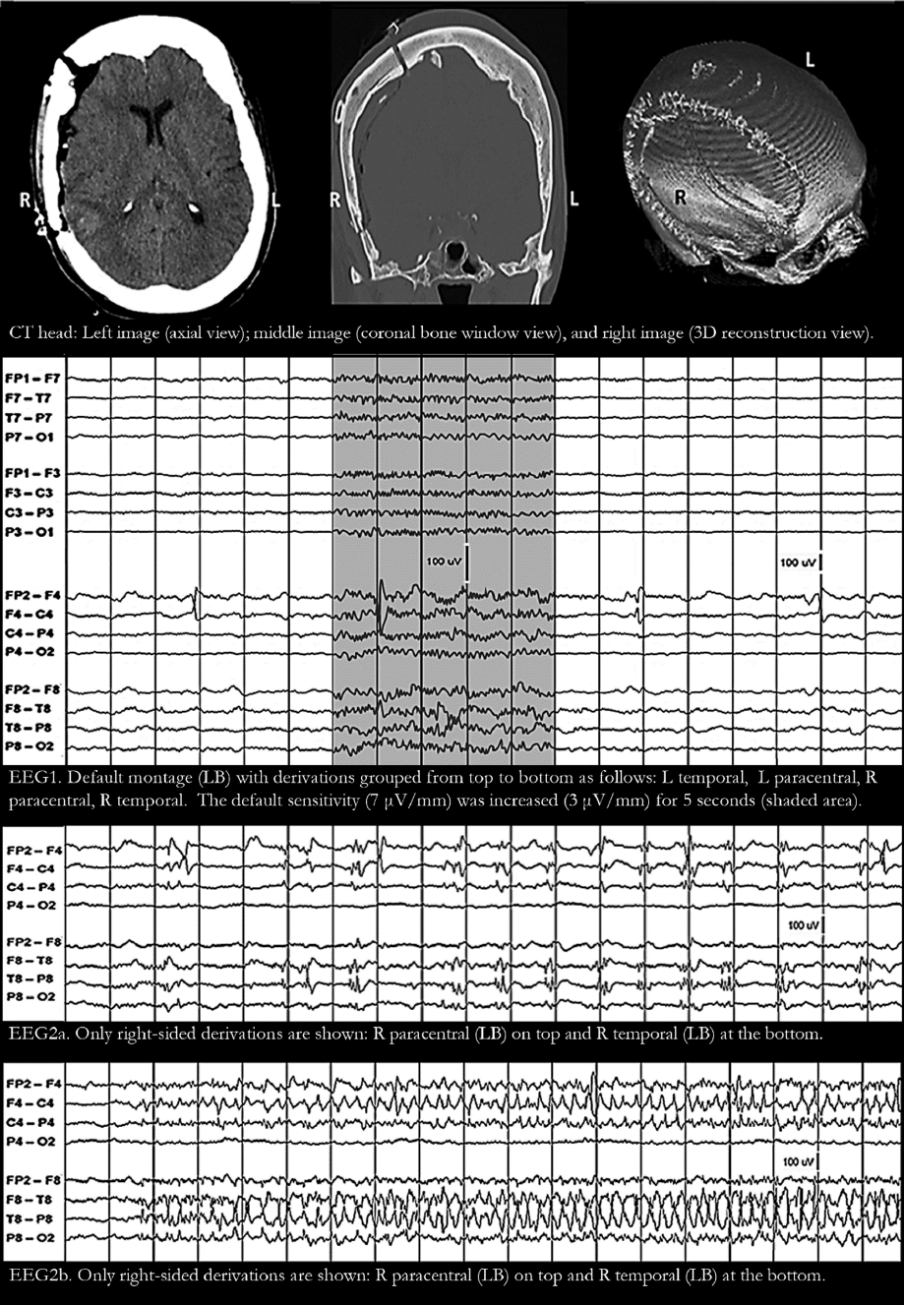
Patient 3 has a large right frontotemporal craniectomy with bone flap in place (CT [computed tomography] head). Electroencephalogram (EEG) showed intermittent F4 potentials with epileptiform morphology but no physiological field suggesting F4 electrode artifacts (EEG1). Increasing display sensitivity showed a hint of a physiological field but this was not enough to rule out electrode artifact (EEG1 shaded area). Adding 10-10 electrodes was not necessary since focal periodic epileptiform discharges (EEG2a) and focal seizures (EEG2b) were subsequently recorded in F4 and T4. For all EEG tracings above: filters at 1-Hz high-pass, 70-Hz low-pass, and 60-Hz notch; 1 second/division; and 100-µV voltage scale shown in tracing. L, left; R, right; Ref, reference; LB, longitudinal bipolar; TB, transverse bipolar; LR, longitudinal referential; TR, transverse referential.

Patient 3 is a 77-year-old woman who became unresponsive after falling at home and hitting her head on the floor. Her right pupil was dilated and non-reactive on arrival, so intubation was immediately performed. CT head revealed a large right subdural hemorrhage and emergency evacuation was achieved with a right hemicraniectomy followed by bone flap replacement (Figure 3: CT head). Postoperatively, she developed recurrent focal motor seizures of the left face and left arm. EEG monitoring initially showed intermittent F4 potentials with epileptiform morphology but without clear-cut “physiological field”—even when display sensitivity was increased from 7 to 3 µV/mm (Figure 3: EEG1). Once again, the absence of a physiological field raised the possibility of F4 electrode artifacts. The EEG subsequently showed periodic epileptiform discharges and focal seizures in F4 and T4 (Figure 3: EEG2-3). With such evidence of right frontotemporal cortical hyperexcitability and epileptogenic focus, it would be impractical to add 10-10 electrodes and extend the montage. Thus, the same issue was virtually resolved in patient 3, not by adding electrodes and extending the montage, but through hindsight (our experience with the first 2 patients) and by taking other findings into consideration (focal periodic epileptiform discharges and focal seizures in F4 and T4).

## Discussion

In all 3 patients, in whom the F4 electrode was directly above or near a skull defect, the scalp EEG showed phase-reversing waves in FP2-F4 and F4-C4 with no clear-cut “physiological field” (even when the EEG was displayed at a higher sensitivity). Cleaning the scalp thoroughly, replacing the F4 electrode, and maintaining electrode impedance between 2 and 5 kΩ failed to eliminate the “electrode artifacts” from the EEG in patients 1 and 2. However, when the EEG was recorded from four 10-10 electrode sites around F4, it became clear that the F4 phase reversals were focal epileptiform discharges, not electrode artifacts. Correlation with other electroclinical and neuroimaging data was enough to resolve this issue in patient 3.

Scalp EEG voltage and spatial rate of change depends on the activity of the cortical generators, the position of the electrodes relative to the generators, and the electrical properties of the conductive media.^4,5^ The latter include the brain, cerebrospinal fluid, meninges, skull, and skin.^16^ With its high resistivity, the skull has a huge impact on the voltage distribution of EEG signals on the scalp—it acts as a spatial filter that results in smoothing and attenuation of the EEG signal.^3,6^ Thus, in humans with intact skull (neonates and infants excluded), cortically generated EEG signals that reach the surface of the scalp usually appear as scalp electrical potentials with a “physiological field.”^14,15^ A skull defect can modify the EEG voltage distribution on the scalp. Since a hole in the skull provides a low-resistance path where EEG signals can pass through with minimal signal attenuation and smoothing, a craniotomy or craniectomy will allow us to see EEG signals with a wider range of frequencies, enhanced power at higher frequencies, and better interelectrode spatial resolution.^3^ Most important, a skull defect can increase the intensity of scalp potentials and result in a compact field distribution. Due to the compact field configuration, the EEG obtained from a standard array of 10-20 electrodes may show cortically generated signals as phase reversals without a physiological field, analogous to the pseudo electrode artifacts in the EEG of the patients we presented, which were later found to be epileptic discharges. Adding extra 10-10 electrodes and displaying the EEG using an extended montage can help clarify the presence of a physiological field.

The effect of a skull defect on the EEG is usually discussed in books and courses in the context of the breach rhythm.^17-19^ For example, it is often pointed out that mu and beta rhythms can resemble epileptiform spikes when there is a skull defect.^20^ However, a hole in the skull can also modify the appearance of EEG waves in ways that are less familiar to EEG professionals. As described in this article, focal epileptic activity underneath a skull defect may mimic electrode artifacts with symmetric phase reversals in 2 adjacent channels and without a physiological field on a standard 10-20 bipolar montage. It is important to be aware of this potential pitfall because continuous EEG after head trauma or brain surgery is now commonplace. This issue can often be resolved technically by adding 10-10 electrodes and extending the montage or by simple correlation with available neuroimaging results and other electroclinical findings.

## Conclusion

What appeared to be F4 electrode artifacts in the EEG of 3 patients with a skull defect were actually focal F4 epileptiform discharges with a steep voltage drop-off. EEG professionals should be aware that focal epileptic activity underneath a skull defect may appear on a standard bipolar montage as electrode artifacts—waves with no physiological field and with phase reversals in 2 adjacent channels. This issue can be resolved by extending the montage based on the 10-10 system and by taking neuroimaging and other electroclinical findings into consideration.
